# Port-Wine Stains and Intraoral Hemangiomas: A Case Series

**DOI:** 10.7759/cureus.63532

**Published:** 2024-06-30

**Authors:** Ignatious Jeba Mary R, Ezhilarasi Arumugam Venkatachalam Sargurunathan, Ramachandra Reddy Gowda Venkatesha, Karthik Rajaram Mohan, Saramma Mathew Fenn

**Affiliations:** 1 Oral Medicine and Radiology, Vinayaka Mission's Sankarachariyar Dental College, Vinayaka Mission's Research Foundation (DU), Salem, IND

**Keywords:** trigeminal nerve, capillary hemangioma, buccal mucosa alterations, gingiva, port-wine stain

## Abstract

Port-wine stains (PWSs), also called port-wine birthmarks or nevus flammeus, are hamartomatous vascular capillary malformations that clinically appear as erythematous areas on the buccal mucosa, vermilion border of the lip, gingiva, or pink to port-wine-colored patches on skin since birth and persist throughout life. On the face, they occur in the area supplied by the trigeminal nerve. PWSs have structural abnormalities of the intradermal capillaries. PWSs on the skin and oral mucosa contain ectatic capillaries in the dermis and submucosa, respectively. PWSs occur anywhere, and the oral mucosa is no exception. PWSs on the facial skin lead to cosmetic disfigurement and create social stigma. Clinically, PWSs start as flat, pink, or red patches and may darken, thicken, and develop nodules over time. The diagnosis of PWSs is primarily clinical. PWSs are complex vascular malformations with significant clinical, psychosocial, and therapeutic challenges. This article enlightens a series of cases of PWSs on the facial skin and capillary hemangioma on the gingiva, buccal mucosa, and lip diagnosed by a diascopy test, etiopathogenesis, differential diagnosis, and management of PWSs.

## Introduction

The capillary vascular malformation occurring in the dermis of facial skin is also known as "port-wine stain" or "nevus flammeus" and is usually seen in the territory of the trigeminal nerve in Sturge-Weber syndrome (SWS) [1]. A port-wine stain (PWS), often known as a fire mark, is a permanent pink or erythematous macular telangiectasic pigmentation that is present from birth and persists throughout a person's life. PWSs are named for their resemblance to the deep purple color of Portuguese fortified red wine [2]. The skin of the forehead, temple, and cheek lateral to the ala of the nose, supplied by the first division of the trigeminal nerve (V1) and second division of the trigeminal nerve (V2) dermatomes, are the most frequently affected areas. Sometimes the affected areas also include the trunk and limbs. The change in color is a result of an increase in the amount of hemoglobin in the skin, caused by the widening of small blood vessels called post-capillary venules and capillaries in the affected areas. The condition affects both female and male babies, occurring in around 50,000 to 1 in 20,000 live births [2]. It can be either localized or extensive, including the deeper blood veins of the skin and underlying tissues. The condition affects around 0.3-0.5% of newborn babies [2]. Researchers have demonstrated that an activated somatic mutation causes PWSs, although the exact etiology remains unclear. Researchers have identified a mutation in the guanine nucleotide binding protein subunit alpha q polypeptide (GNAQ), specifically the 548G (guanine nucleotide binding protein) variant as the cause of this condition [2,3]. The mTOR signaling pathway exhibited heightened activation in advanced PWSs. Increased activation of the mTOR signaling pathway may play a role in the enlargement and formation of nodules in PWSs. The findings offer initial support for the therapeutic potential of targeting the mTOR signaling system in the treatment of PWSs and associated diseases [4].

## Case presentation

Case one

**Figure 1:**
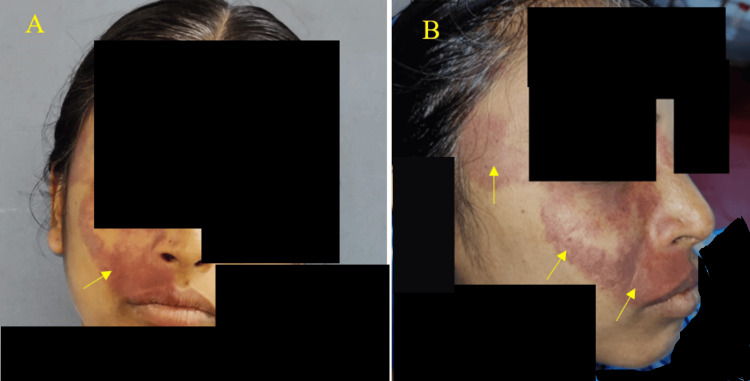
A. Presence of unilateral purplish flat discolored macular areas on the right side of the face lateral to the right ala of the nose, philtrum of the vermilion border of lip not crossing the midline (yellow arrow). B. Port-wine stains on the right side of the face (yellow arrows).

A 37-year-old female patient came to our outpatient department of oral medicine and radiology for a routine dental checkup. A general examination revealed her vitals were stable. There was no history of harmful chewing or smoking tobacco. During the extraoral examination, a port-wine-colored macular patch, characterized by its irregular shape and margins, was only visible on the right side of the face. The macular patch extends superiorly from the right temple, close to the level of the right eyebrow, and extends inferiorly to the vermilion border on the right side of the upper lip. The macular patch extends anteriorly from the right lateral ala of the nose and extends posteriorly 2.5 cm from the tragus of the right ear (Figures 1A, 1B).

**Figure 2:**
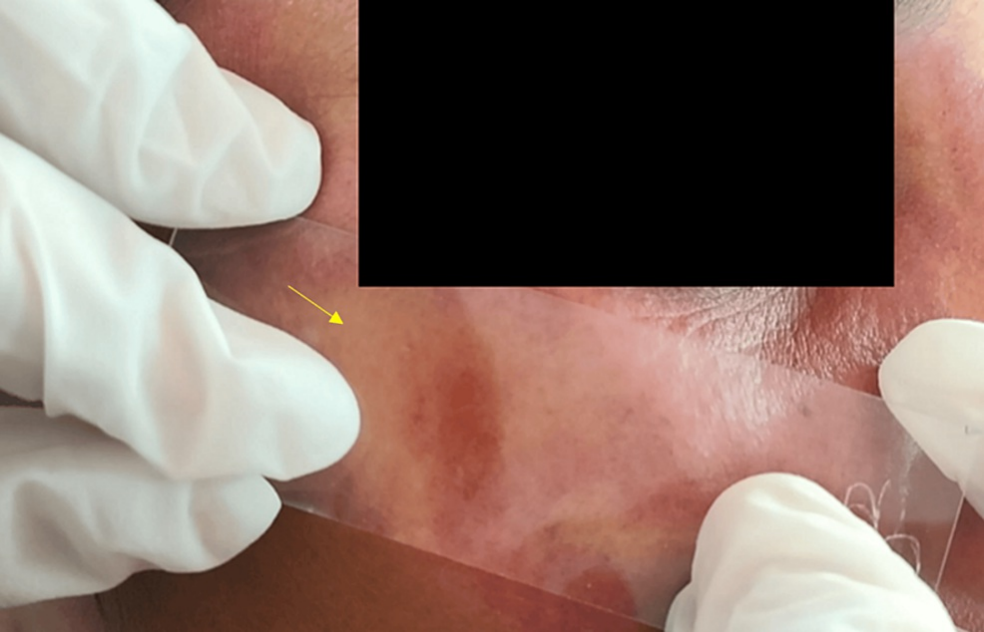
Diascopy test on the port-wine-colored macular patches using a microscopic glass slide on the skin near the malar region showed positive blanching (yellow arrow).

History reveals they were present from birth. We observed no similar lesions elsewhere in the body. Her medical history was non-contributory. The patient reported no history of seizures or blurred vision. The diascopy test performed on the port-wine-colored macular patches showed positive blanching results (Figure 2).

**Figure 3:**
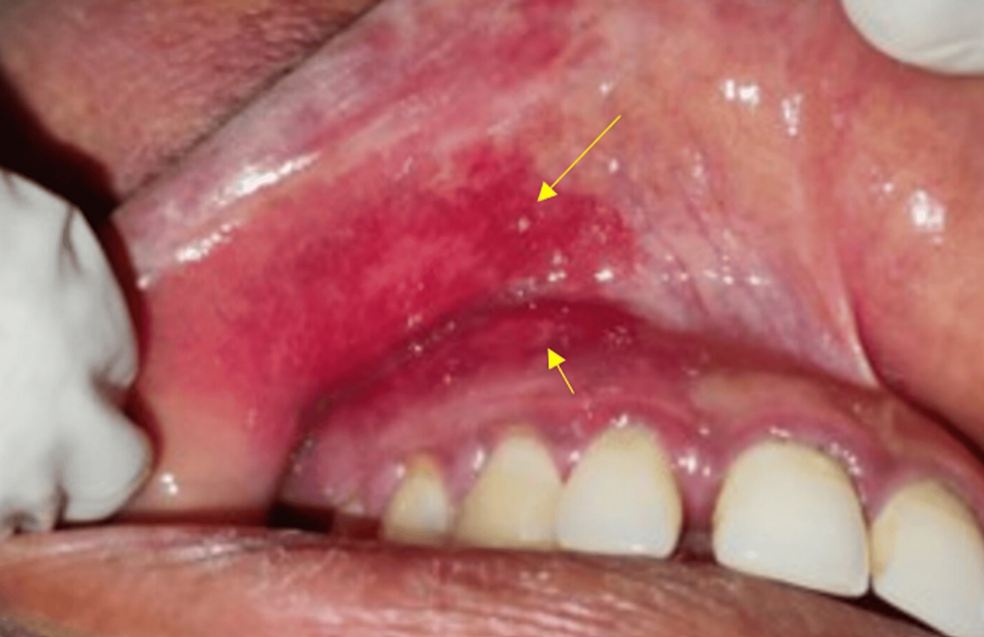
Intraoral examination revealed bright fiery-red areas involving labial mucosa and attached gingiva in relation to right maxillary lateral incisor, canine, and premolar regions (yellow arrow).

During the intraoral examination, we noticed a bright, fiery, red-colored maxillary gingiva on the right side, adjacent to the buccal mucosa (Figure 3).

**Figure 4:**
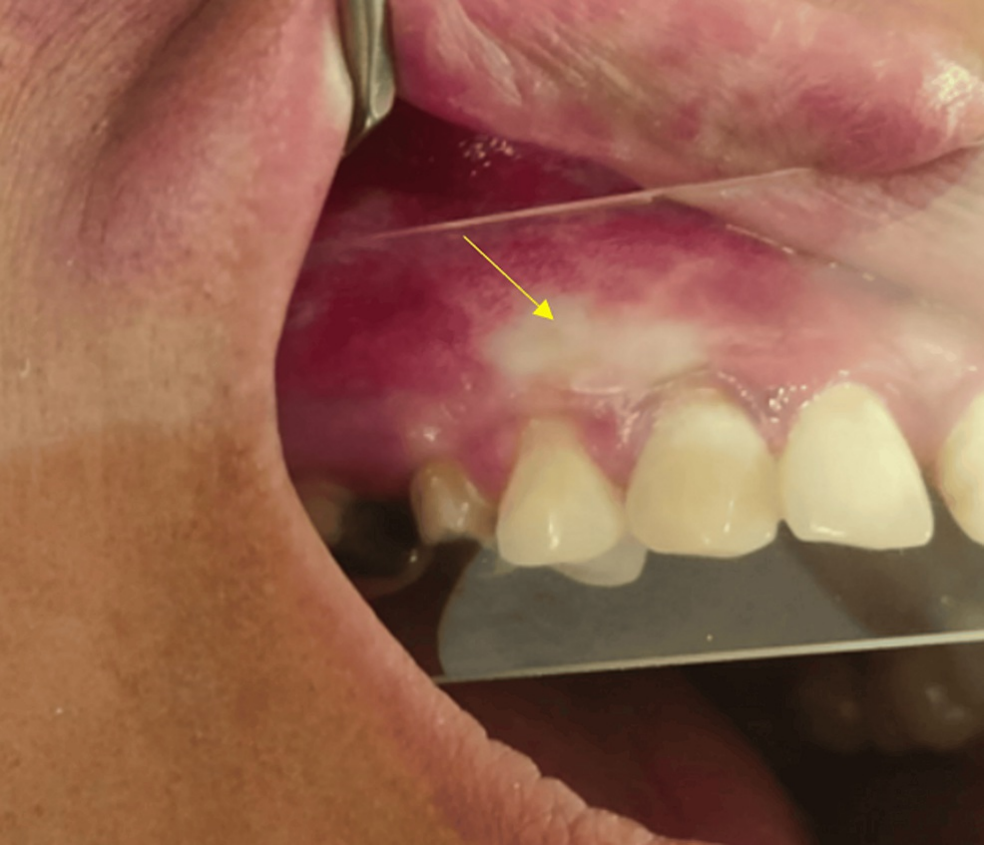
Diascopy revealed blanching of attached gingiva (yellow arrow). Diascopy test performed by gentle pressure using a microscopic glass slide on the attached gingiva showed blanching.

The diascopy test was done chairside by applying gentle pressure to the bright, fiery-red areas on the attached gingiva in relation to her right maxillary canine and first premolar teeth, which revealed blanching (Figure 4). Hence, the diascopy test was considered positive and confirmed the presence of vascular malformations, namely, capillary hemangiomas, in these areas.

The demonstration video of the diascopy test performed on the gingiva is shown in Video 1.

**Video 1:** Diascopy test. The diascopy test performed by the application of pressure from the microscopic glass slide on the surface of the attached gingiva revealed blanching (positive diascopy test)

The purple-colored macular patches were consistent with the trigeminal nerve distribution. They were only on the right side of the face, following the first, namely the ophthalmic division of the trigeminal nerve (V1), and the second, the maxillary division of the trigeminal nerve (V2) branches of the dermatome supplied by the right trigeminal nerve. The patient had calculus in her mandibular anterior tooth region due to inadequate dental care. The positive diascopy test provisionally diagnosed a PWS involving the facial skin and gingiva. The differential diagnosis for this case includes pyogenic granuloma, which clinically occurs as a reactive growth in response to localized supra and sub-gingival calculus and bleeds on slight provocation. We performed an oral prophylaxis and referred the patient to a dermatologist for the management of PWSs on her face.

Case two

**Figure 5:**
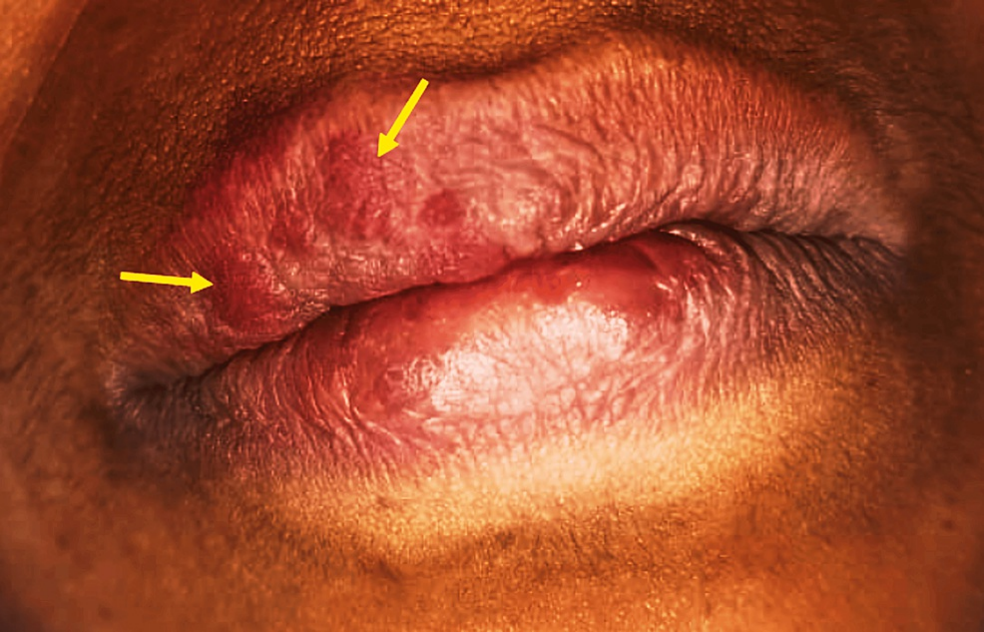
Extraoral examination revealed discrete port-wine-colored areas only on the right-side vermilion border of the upper lip not crossing the midline (yellow arrow).

A 45-year-old female reported to our department for a routine dental checkup. Upon extraoral examination, a discrete purplish enlargement was only visible on the right-side vermilion border of her upper lip, not extending across the midline (Figure 5).

**Figure 6:**
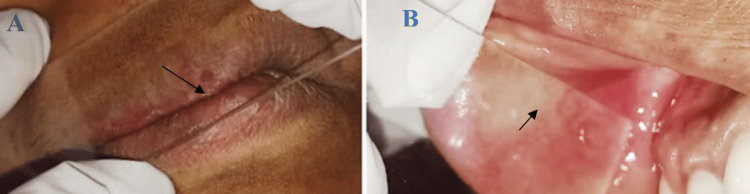
A. Diascopy test showed blanching on the right-side upper vermilion border of the lip. B. Diascopy test showed blanching of the right buccal mucosa (black arrow).

The intraoral examination revealed fiery reddish areas of the gingiva and right buccal mucosa, which blanched on pressure application (Figures 6A, 6B).

**Figure 7:**
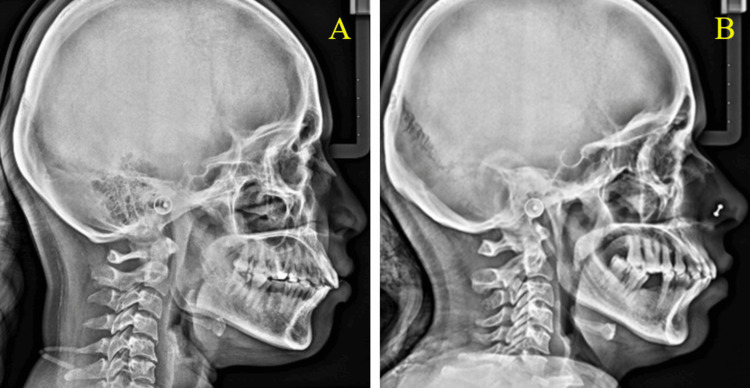
Lateral cephalometric radiograph did not reveal any calcifications.

The port-wine-colored macular areas are confined only to the right side of the facial skin. The maxillary division of the trigeminal nerve (V2) supplies the port-wine-colored macular areas only to the right-side vermilion border of the upper lip and the bright-red macular areas on the ipsilateral right buccal mucosa. The patient was referred to a dermatologist for treatment. We advised a lateral skull radiograph in both cases to rule out the possibility of any intracranial calcification associated with syndromes like Sturge-Weber syndrome. The lateral cephalometric skull radiograph showed no calcification (Figures 7A, 7B).

The general differential diagnosis for PWSs or nevus flammeus includes rare congenital syndromes involving capillary abnormalities and pigmented lesions, with or without systemic symptoms, called phacomatosis pigmentovascularis. It was classified into four phenotypes (I: Nevus flammeus and nevus verrucous or pigmentosus; II: flammeus +/- anemic and Mongolian spots; III: flammeus +/- anemic and spilus; IV: flammeus +/- anemic and spilus with Mongolian spot) and subtypes. Happle recharacterized it as phacomatosis, cesioflamea, cesiomarmorata, spilorosea, and unclassified without or with systemic symptoms [5]. Thus, a diagnosis of intraoral hemangioma with a PWS on the right side of the face was made. We referred her to consult a dermatologist for further treatment of the PWS. Informed consent was obtained in both the above-mentioned cases.

## Discussion

Nevus flammeus, or port-wine stain, is a congenital non-neoplastic capillary hamartomatous malformation affecting the capillaries in the dermis that causes a pink, crimson, or port-wine color patch on the skin of newborns and persists throughout life. The distorted capillary-like capillaries form the well-defined, unilateral, bilateral, or centrally positioned pink to crimson nevus flammeus on the face during birth [2].

The cause of nevus flammeus has not been fully determined. The majority of cases are sporadic, while there have also been reported instances of familial cases [2]. The progress achieved in the study of genetics has played a crucial role in identifying the genetic foundation of both non-syndromic and syndromic types of nevus flammeus. A mutation in the GNAQ is found on chromosome 9 at location 9q21.2 and has been observed in both isolated nevus flammeus and SWS [2].

Dysregulation of the neurons and excessive expression of vascular endothelial growth factors and their components have also been suggested as causative causes for the development of nevus flammeus [3]. Occasionally, trauma can lead to the development of a rare condition called "Fegeler syndrome," which is characterized by the presence of an acquired nevus flammeus.

A PWS is a ubiquitous type of vascular anomaly. It often manifests from birth, impacting approximately 0.3% to 0.5% of infants, frequently in the craniofacial area. There is no observed preference based on gender. Understanding the molecular profile of blood vessel endothelial cells, the ultrastructural alterations in affected skin, and the genetics of vascular malformations have helped us understand this congenital skin disorder [3]. 

This vascular malformation may be caused by congenital vessel wall weakening, perivascular dermal defects, neuromodulation disorders, and cephalic neuroectoderm dysmorphogenesis [3]. A congenital vascular or capillary malformation is nevus flammeus. Adults with persistent lesions may have subpapillary capillary ectasia. Vascular endothelial growth factor may stimulate angiogenesis and vasodilation in nevus flammeus [3].

Recent investigations support the two main hypotheses: genetic abnormalities and faulty nerve innervations to dermal blood vessels dysregulate angiogenic signaling. The considerable decrease in cytosolic proteins soluble in 100% ammonium sulfate (S-100) positive nerve fibers in dermal vasculature shows that PWS vessels lack normal nerve innervation. PWSs result from vascular basal tone decline and/or neurotrophic factor depletion [3]. It has been suggested that embryologically faulty sympathetic fiber development in dermal vessels causes sympathetic control loss and vascular ectasia [3].

PWS dermal blood vessels coexpress arterial marker Ephrin (EfnB2) and venous marker Ephrin (EphB1), resulting in aberrant differentiation of the primary capillary plexus into dermal venules and arterioles. A gradual dilation of the vasculature results in venule-like results. Abnormal activation of angiogenesis-related mitogen-activated kinase-like protein and phosphatidylinositol 3-kinase (PI3K) signaling pathways has also been linked [3]. Mutations in GNAQ, RAS p21 protein activator 1 (RASA1), and Ephrin (EphB1/EfnB2, and EphB4) coexpression cause aberrant activation. The causes of aberrant and dilated blood vessels include the following: Somatic GNAQ (R183Q) mutations induce angiopoietin-2, increase capillary-like vessels, and produce fewer perivascular nerves, Ephrin B2 and B1 coexistence, lack of alpha-smooth muscle actin, new mutations in SWItch/Sucrose non-fermentable (SWI/SNF) related, matrix associated, actin dependent regulator of chromatin, subfamily a, member 4 (SMARCA4), Ephrin (EPHA3), phosphatidylinositol-4,5-bisphosphate 3-kinase catalytic subunit alpha (PIK3CA), myeloblastosis proto-oncogene, transcription factor (MYB), and platelet-derived growth factor receptor beta (PDGFR-beta) [3].

PWSs across the midline lumbosacral region may indicate concealed spinal dysraphism. When PWSs are coupled with a hemangioma, tuft of hair, dermoid cyst, lipoma, or true/pseudo tail, imaging is recommended [3]. Fegeler characterized trauma-related skin lesions that showed morphological and histological similarities to PWSs in 1949. Fegeler syndrome, or acquired PWS, is a rare clinical condition that responds better to PDLs [3]. Trauma is the most common trigger; however, accutane, oral contraceptive pills, metformin, simvastatin, frostbite, herpes zoster, and peritoneovenous shunt blockage have all been observed [3]. 

PWSs, the most prevalent vascular malformations, appear as pink or red homogeneous, variable-sized macules and patches with borders geographically from birth that last a lifetime. There is no discomfort, no spontaneous bleeding, no warmth. It affects the skin of the neck and head in 70% to 90% of cases, but it can also affect the trunk or extremities and, infrequently, the mucosa [3].

Phacomatosis cesio-flammeo-marmorata is a rare association of nevus flammeus on the face, aberrant Mongolian spots, and congenital telangiectatic cutis marmorata [6]. The Renbok phenomenon (inverse Koebner phenomenon) occurs in nevus flemmeus [7].

SWS is categorized as complete when it affects both the central nervous system and angiomas on the face and incomplete when it affects only one [8]. The Roach scale classifies SWS into three types: Type I, which has both facial and leptomeningeal angiomas and may also have glaucoma; Type II, which has only facial angiomas and may also have glaucoma; and Type III, which has only leptomeningeal angiomas and no glaucoma [8]. The patient's condition corresponds to Type II on the Roach scale in both our cases.

Lesions occur single or numerous, bilateral or unilateral, widespread or localized. PWSs show segmental distribution over the face, following the trigeminal nerve. Lesions deepen and turn purple with age. Progressive dermal vascular ectasia causes face PWSs to thicken and hypertrophy later in life, with or without nodularity. Facial PWS patients rarely develop fat, muscle, and bone hyperplasia [8,9]. 

PWSs may be part of a genetic syndrome in people with various congenital impairments or as an independent vascular abnormality such as klippel-trenaunay Weber, mafucci, and sturge-weber syndromes. Additionally, it has been suggested that there may be a malfunction in the embryological development of the sympathetic fibers, leading to a disruption in the normal control of the blood vessels in the skin, which in turn causes ectasia [10].

PWSs, also known as nevus flammeus, are congenital vascular abnormalities that appear as red birthmarks near the nose and forehead. The trigeminal nerve branches, namely the first division of trigeminal nerve (V1), second division of trigeminal nerve (V2), and third division of trigeminal nerve (V3), are superimposed on top of each other and persist throughout the lifetime of an individual [10]. About 87-90% of patients have PWSs, usually on the right. These lesions might occur on both sides in 33% of cases [10]. 

The stigma of PWSs, especially over the face, can lower self-esteem and stress. Multiple problems and comorbidities can accompany combined vascular malformations and syndromes. Successful therapy requires discussions and professional counseling with multiple medical specialists, such as dermatologists, interventional radiologists, and vascular specialists, to assist both the family and patient in overcoming challenges and improving the prognosis [8].

The patient had a widespread reddish-purplish patch (PWS) on the right side of the face, specifically in the regions innervated by the largest cranial nerve, namely the trigeminal nerve. PWSs were present from birth in both cases. 

The anatomical position of a vascular malformation is associated with an increased likelihood of developing glaucoma, leptomeningeal calcifications, and angiomas [7]. However, glaucoma and leptomeningeal calcifications were not seen in any of our cases. Pyogenic granuloma is a single ulcerated lump on a PWS that bleeds with minor trauma. Adults can develop them spontaneously after trauma, pregnancy, or the use of an oral contraceptive pill. Individual cases of PWS-tufted angioma have been reported [8].

Similar to the Meyerson phenomenon, studies have described severe eczema or PWS-specific eczema [9]. Oral vascular lesions can range from an individual increase in blood vessels to a significant growth of blood vessels or the formation of pyogenic granulomas. As a result, the mucosa becomes delicate and susceptible to even minor injuries and simple dental operations. The lesions exhibit a distinct center boundary and are located on the same ipsilateral side as the port-wine lesions on the face [9]. We attribute the gingival redness in our patient to the presence of angiomatous growth and inadequate oral hygiene.

Isolated PWSs are benign and usually just cosmetically significant [9]. The existence of a PWS causes significant emotional distress for the patient, and it affects the formation of personality in almost all people. Possible methods to enhance the appearance of PWS include dermabrasion, tattooing, and the use of pulsed diode lasers (PDLs) [9]. PDL treatment is more challenging for darker skin types: light-brown skin, which rarely burns and tans easily (Fitzpatrick IV), brown skin, which never burns and tans very easily (Fitzpatrick V), and black skin, which is heavily pigmented because it never burns and tans very easily (Fitzpatrick-VI) due to the inverse relationship between PDL vascular specificity and skin pigmentation, which requires caution when treating darker skin types with PDLs [10].

Early laser therapy produces better results since age-related thickness and nodularity make it resistant to treatment [10]. The flash lamp PDLs were the pioneering laser designed exclusively for the treatment of PWSs [11]. The laser initially had a wavelength of 577 nm, which was chosen to align with the third peak of the absorption curve of hemoglobin. It also had a pulse duration of 0.45 milliseconds, which was less than the transient relaxation time (TRT) of the smaller capillaries (postcapillary venules) in PWS. Subsequently, it was substituted with yellow light at wavelengths of 585 and 595 nm, which had increased depth of penetration and yielded superior outcomes. The turnover time of the epidermis ranges from 3 to 10 milliseconds. The capillaries of PWSs are extremely narrow, measuring between 50 and 100 µm in diameter, and have a transient response time ranging from 1 to 5 milliseconds. Unless the pulse is extremely brief, it cannot be heated to the point of destruction. The pulse width of PDLs is 0.45 milliseconds, which is less than the TRT of the smaller blood vessels in PWS. In addition, the epidermis is safeguarded by the dynamic cooling device. While the PDL is commonly regarded as the preferred treatment for most cases of PWSs, intense pulse light (IPL) sources with a cutoff filter of 590 nm are also highly effective and are regularly employed for many superficial vascular diseases, including PWS [11]. The PDL using 595 nm filters at 3.5 millisecond double pulse and thermal relaxation time and 20 millisecond time delays following 10 full and 3 touch-up treatments provided effective resolution of PWSs on 50-100 micrometer-diameter vessels. The adverse effects include subepidermal peeling and purpura following PDL therapy [11].

Rarely, mucosal involvement causes gingival hyperplasia, which can lead to periodontal disease and poor tooth hygiene. PDL-caused skin barrier degradation in PWSs was alleviated by an innovative glycyrrhizic acid dressing [12]. IPL and a 585 nm long-pulsed diode laser may be more effective than a short-pulsed diode laser for treating recalcitrant Port wine staining [13].

A refined DeepLabV3+ network (IDeepLabV3+) was created specifically for the purpose of segmenting PWS lesions. This network was built around the foundational architecture of the original DeepLabV3+ model [13]. The deep learning-based semantic segmentation technique can intelligently divide PWS lesions of varying color and shape in the texture mapping of three-dimensional (3D) pictures [14]. Semantic segmentation can be employed in conjunction with 3D scanning to assess the size of face PWS lesions [14].

Recently, various non-invasive diagnostic methods have been used to examine PWSs. These techniques include dermoscopy, VISIA-CR™ system, reflectance confocal microscopy (RCM), high-frequency ultrasound (HFUS), optical coherence tomography (OCT), photoacoustic imaging, laser scatter imaging (LSI), and laser Doppler imaging (LDI) [15].

Currently, dermoscopy is a straightforward and precise method for objectively evaluating the kind of vascularization in PWSs. The VISIA-CR™ systems are crucial for situations when recognizing erythematous changes is difficult without the aid of technology. Nevertheless, they lack the capability to identify vessels located at great depths. On the other hand, RCM is distinguished by its ability to provide detailed and clear images, allowing for the evaluation of skin blood vessels, blood flow, and flow speed. Nevertheless, its functionality is restricted to the surface layer of the skin, which hampers the evaluation of deep blood vessels. On the other hand, HFUS is a highly efficient instrument for assessing the thickness and depth of profound lesions in PWSs [15].

OCT is a rapid and precise method for capturing small alterations in tissue structure. There are other advanced examination methods, including Doppler OCT, dynamic optical coherence tomography (D-OCT) used to assess the depth and diameter of blood vessels located up to 1.5 to 2 mm, and optical coherence tomography angiography (OCTA), which can be used to evaluate different types of PWSs more correctly. These methods offer a more accurate evaluation of the vascular information of PWSs, aid in diagnosing the specific pathological kind of PWS, and help in choosing the most suitable therapeutic treatment strategy. Furthermore, photoacoustic imaging is distinguished by its exceptional ability to penetrate deep into tissues and provide a strong contrast between different structures. Some examples of these techniques are photoacoustic microscopy and photoacoustic dermoscopy. The former enables the investigation of microvascular structures without causing any harm, while the latter can accurately show detailed structures from the outermost layer of the skin to the layers beneath it. This compensates for the natural limits of high-frequency skin ultrasonography. The use of PDL therapy for conditions such as PWSs, LSI and LDI, has demonstrated its efficacy in monitoring blood perfusion [15]. Dev et al. (2014) stated that topical agents such as timolol, imiquimod and rapamycin may be used in combination with laser therapy but are ineffective for PWSs with rapid hypertrophy and nodularity. In such cases, sclerotherapy using sodium tetradecyl sulfate under fluoroscopy guidance is beneficial to treat a PWS with rapid hypertrophy and nodularity [16]. Rao diagnosed a PWS on the lower lip by diascopy [17]. Adult and pediatric hypertrophic and treatment-resistant PWSs can be treated using the Alexandrite 755 nm laser [18].

Photothermal transduction agents (PTAs) capture and transform energy from light of a certain wavelength into heat, resulting in an elevation of the temperature in the surrounding microenvironment. Cell death would occur fast at temperatures ranging from 46 to 52 °C as a result of microvascular thrombosis and ischemia. PTAs can be categorized into two groups: inorganic materials, such as graphene, carbon nanotubes, and boron nitride, and organic materials, specifically NIR-responsive small molecules. Usually, PTA absorptions are calibrated within the range of 750 to 1350 nm in order to align with the tissue-transparent window. As a result, certain researchers attempt to utilize photothermal therapy (PTT) as a treatment for individuals with PWSs because there is a chance that PTT may have therapeutic benefits on the inner blood vessels. An investigation unveiled that the absorption of blood amplified by a factor of 3.9 at a wavelength of 1064 nm subsequent to the administration of gold polyethylene glycol (PEG)-modified gold nanorods into the bloodstream. Additionally, the minimum energy density required for the therapy of PWSs was lowered by 33%. In a separate investigation, the vessels that had near-infra-red fluorescent (NIR) erythrocyte-derived transducers and were exposed to 585 nm irradiation exhibited elevated levels of photothermal damage compared to the vessels that did not have these transducers. The aforementioned findings indicate that PDL holds promise as a viable treatment for individuals with PWSs. To enhance the precision of targeting blood vessels in PTAs, it is advisable to combine PTAs with surface markers for the treatment of PWSs [19].

PDL continues to be the benchmark for PWS treatment; however, the rate of recurrence is significant. Topical medications including imiquimod, axitinib, and rapamycin, when used in combination with PDL therapies, have the potential to modify recurrence rates and decrease the number of PDL sessions required for individuals with PWSs. The use of nanocarriers coupled to surface markers, such as monoclonal antibodies and peptides, encapsulating photosensitizers, or PTAs, shows promising therapeutic promise in PDL or PTT for PWSs targeting the deep vascular plexus [19].

The combination of 0.5%-1% sirolimus (SIRO) cream and PDL, along with the use of a non-laser skin resurfacing technology called Tixel, is employed to address the challenges posed by the limited bioavailability of the medicine. As a result, alternative methods to increase permeability may be a viable choice for treating deeper or more hypertrophic forms of PWSs [18].

## Conclusions

Diascopy is a simple potential chairside examination for the diagnosis of PWSs. It entails gentle application of pressure on suspected PWSs on facial skin or capillary vascular malformations like gingiva or buccal mucosa hemangiomas and monitoring blanching with a microscopic glass slide. Due to its simplicity and non-invasiveness, diascopy is recommended for first-line diagnosis. This has major clinical implications for the diagnosis of PWSs.
